# Identifying the outcomes important to men with hypogonadism: A qualitative evidence synthesis

**DOI:** 10.1111/andr.13156

**Published:** 2022-02-08

**Authors:** Magaly Aceves‐Martins, Richard Quinton, Miriam Brazzelli, Moira Cruickshank, Paul Manson, Jemma Hudson, Nick Oliver, Rodolfo Hernandez, Lorna Aucott, Frederick Wu, Waljit S. Dhillo, Siladitya Bhattacharya, Katie Gillies, Channa N. Jayasena, NIHR Testosterone Efficacy & Safety (TestES) Consortium

**Affiliations:** ^1^ Health Services Research Unit University of Aberdeen Aberdeen UK; ^2^ Translational & Clinical Research Institute University of Newcastle‐upon‐Tyne Newcastle upon Tyne UK; ^3^ Department of Endocrinology Diabetes & Metabolism Newcastle‐upon‐Tyne Hospitals NHS Foundation Trust Newcastle upon Tyne UK; ^4^ Department of Metabolism Digestion and Reproduction Faculty of Medicine Imperial College London London UK; ^5^ Health Economics Research Unit University of Aberdeen UK; ^6^ Division of Diabetes Endocrinology & Gastroenterology Manchester Institute for Collaborative Research on Ageing School of Social Sciences University of Manchester Manchester UK; ^7^ Aberdeen Maternity Hospital School of Medicine University of Aberdeen Aberdeen UK

**Keywords:** male hypogonadism, qualitative systematic review, testosterone replacement therapy

## Abstract

**Objective:**

Men with male hypogonadism (MH) experience sexual dysfunction, which improves with testosterone replacement therapy (TRT). However, randomised controlled trials provide little consensus on functional and behavioural symptoms in hypogonadal men; these are often better captured by qualitative information from individual patient experience.

**Methods:**

We systematically searched major electronic databases to identify qualitative data from men with hypogonadism, with or without TRT. Two independent authors performed the selection, extraction, and thematic analysis of data. Quality of eligible studies was assessed using the Critical Appraisals Skills Programme and Grading of Recommendations Assessment, Development and Evaluation‐Confidence in the Evidence from Reviews of Qualitative research tools.

**Results:**

We analysed data from five studies published in nine reports that assessed a total of 284 participants. Published data were only available within North America, with no ethnic minority or other underserved groups included. In addition to sexual dysfunction, men with MH experienced adverse changes in physical strength, perceptions of masculinity, cognitive function, and quality of life. The experience of MH appeared dependent on the source(s) of educational material.

**Discussion:**

We propose a patient‐centred approach to clinician interactions rather than focusing on discreet MH symptoms. Current evidence about the experience of MH is limited to North America and predominantly white ethnicity, which may not be broadly applicable to other geographic regions. Broadening our understanding of the MH experience may improve the targeting of information to patients. In addition, a multidisciplinary approach may better address symptoms neither attributable to MH nor alleviated by TRT.

## 1 INTRODUCTION

Numerous clinical trials have investigated the ability of testosterone replacement therapy (TRT) to alleviate male hypogonadism (MH) symptoms.^1^ There is consensus that MH causes several symptoms that TRT can improve. However, men investigated for MH often complain of a constellation of less specific symptoms, including physical limitations, tiredness, low mood, and reduced cognition.^2^ There is little agreement among clinicians whether these functional and psychological (behavioural) symptoms are indicative of MH and thus likely to be ameliorated by TRT.^3^, ^4^, ^5^, ^6^

Coupled with prevailing concerns highlighted by the US Food and Drugs Administration (FDA) regarding the cardiovascular safety profile of TRT (and the lack of long‐term safety data in men with prostate cancer), men with MH face an uncertain journey from the onset and evolution of symptoms to seeking and establishing a medical diagnosis to the initiation of TRT and subsequent monitoring of therapy (clinical response and adverse effects).^7^, ^8^ In addition to (and/or as a consequence of) the above‐mentioned 'traditional' androgen‐dependent endpoints, MH is likely to disrupt many important aspects of life for affected individuals, including relationships, self‐image, activities of daily living, and health‐related quality of life (HR‐QOL). Such changes are more challenging to assess and tend to receive less attention from clinicians and researchers.^9^ Hence, there is a paucity of substantive research exploring the subjective experience of men with MH.

Unlike clinical research outcomes, patient‐reported outcomes (PRO) provide direct evidence of how patients feel or function.^10^ The importance of PRO data is reflected by their inclusion in recent FDA guidance for designing trials establishing the efficacy of drugs to treat MH.^11^ The Testosterone Efficacy and Safety (TestES) Consortium was commissioned by the Health Technology Assessment Board, National Institute of Health Research, UK (Project reference HTA 17/68) to conduct a comprehensive evidence synthesis of all aspects of healthcare for MH. Herein, we report the qualitative evidence reporting how men experience MH and the impacts on their lives.

## 2 MATERIALS AND METHODS

We developed comprehensive search strategies to identify published papers reporting qualitative data on the perception and experiences of men with hypogonadism and/or those using TRT. An information scientist searched Ovid Medline, Embase, PsycInfo, EBSCO CINAHL, and Proquest ASSIA for papers published from 1992 to February 2020. References of included studies were perused for further relevant papers (Table S1). One review author (MA‐M) screened all titles and abstracts with a randomly selected 10% cross‐checked by a second review author (KG). A third author (CNJ) was consulted when consensus regarding eligibility could not be reached. We focused on primary studies that explored any aspect of TRT for low testosterone from the perspective of men, their partners, or their clinicians. Mixed methods studies were included if qualitative methods and results were reported separately. The population of interest consisted of adult men (>18 years old) diagnosed with hypogonadism, confirmed either by low testosterone levels or by using TRT. Studies restricted to a singular aetiology of hypogonadism (e.g., Klinefelter's syndrome, congenital hypogonadism, prostate cancer, and so forth) were excluded because of the potential of introducing the experience of symptoms unrelated to hypogonadism per se.

Two reviewers (MA‐M. and KG) independently extracted data from the included papers, shared notes, and discussed study findings and interpretations during a series of meetings. Papers were initially organised alphabetically and subsequently grouped under emerging issues and themes. A data extraction form was developed and piloted for this qualitative systematic review. From each included study, we recorded quotes from participants and/or interpretation of findings by study authors irrespective of whether participants' quotes supported it.

We conducted a three‐phase thematic synthesis using both inductive and deductive approaches. First, we closely scrutinised the included studies to identify the main recurring themes and recorded line‐by‐line coding of the qualitative findings of primary studies; next, we organised these 'free codes' (i.e., single quotes) into related areas to construct 'descriptive' themes; finally, if sufficient data were available, we developed an 'analytical' theme.

Two men with hypogonadism were recruited to advise the research team on key issues including verifying the importance of study questions, refining study design, and interpretation of study findings.

Video conferences were held on 27 January 2021 to give clinician members of the study team the opportunity to gain feedback on the study findings from members of the patient panel. Patients were sent simplified versions of the drafted results beforehand and received summary presentations from CNJ. Comments of the patient panel were into the current report.

### 2.1 Assessment of quality

We appraised eligible studies for methodological rigour and theoretical relevance using the Critical Appraisals Skills Programme (CASP) tool.^12^ Included studies were quality‐appraised by one reviewer (MA‐M), with a second review author (KG) checking the completed assessments. Any disagreement was resolved by discussion or referred to a third review author (CNJ).

### 2.2 Confidence in review findings

We used the Grading of Recommendations Assessment, Development and Evaluation‐Confidence in the Evidence from Reviews of Qualitative research (GRADE‐CERQual) approach to assess our confidence in the findings of the thematic synthesis (MAM and KG double coded).^13^

## 3 RESULTS

### 3.1 Sample demographics

**FIGURE 1:**
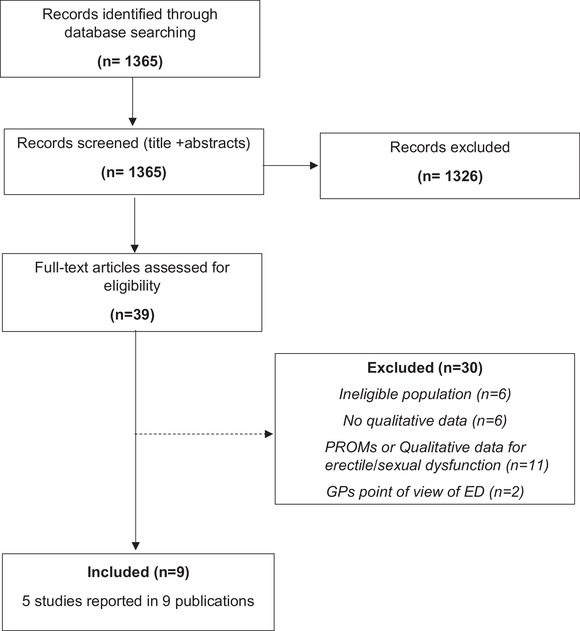
PRISMA flow diagram

The flow diagram of selected studies is shown in Figure 1. Despite comprehensive searches, only five qualitative studies (published in 9 reports) investigating the experience of men with hypogonadism were identified in the literature and deemed suitable for inclusion. Thirty studies were excluded as they did not meet our pre‐specified inclusion criteria. Reasons for exclusion were ineligible populations (six studies), focus on a single symptom of hypogonadism (e.g., erectile dysfunction) (13 studies), or no relevant qualitative data (11 studies). All five included studies were conducted in North America (four in the United States and one in Canada) in men with hypogonadism (284 in total) who were either administered or not administered TRT (see Table 1).^14^, ^15^, ^16^, ^17^, ^18^ One study also reported the perspectives of healthcare providers treating men with hypogonadism.^18^ Participants' age ranged from 18 to 85 years across the studies that reported this information. The diagnostic criteria for hypogonadism were specified in three of the five included studies: Two studies required a total serum total testosterone (TT) level < 300 ng/dl (10.4 nmol/L) as entry criterion; in one study, most participants (22/26) had a total serum TT level <300 ng/dl (10.4 nmol/L) while the remaining participants (4/26) had levels <500 ng/dl.

**TABLE 1:** Participant characteristics of included studies

Study	**Aim (as described within the papers)**	**Condition of focus**	**Participants' characteristics**	**Details of study**	**Qualitative methods**
First author: Gelhorn^14^ Year: 2015 Country: USA	To develop a patient‐reported outcome instrument, the Hypogonadism Impact of Symptoms Questionnaire (HIS‐Q) and to assess its content validity. In a second publication (Gelhorn et al.), authors developed a briefer version of this same tool.^27^	Clinical diagnosis of hypogonadism (total serum TT level <300 ng/el) with or without TRT. The mean of the patients’ lowest recorded testosterone levels was 184.9 ± 55.2 ng/dl, and the patients had been diagnosed with hypogonadism for2.9 ± 3.9 years [range 0.3–20.6] Meantime since diagnosis (clinic report), years (SD) [range]2.7 (2.6) [0.0–11.8]	Sixty‐five male participants, mean age 53.0 [SD 14.1], with hypogonadism (mean serum total testosterone level was 184.9 ± 55.2 ng/dl), could read and speak and understand English. 16.9% were Hispanic or Latino, 83.1% not Hispanic or Latino, race reported as 1.5% American Indian or Alaska Native, 15.4% Black or African American, 75.4% White, 7.7% other, 86.2% were living with partner or spouse, family, or friends.	Participants were recruited through eight clinical sites in the United States. Unclear if the population overlaps Gelhorn et al. The instrument development included a literature review, input from expert clinicians (*n* = 4), and qualitative study, including the first phase with concept elicitation focus groups (5–8 participants each, *n* = 25); individual concept elicitation interviews by telephone (*n* = 5) or face‐to‐face (*n* = 9); and a subsequent phase including personal cognitive interviewing (*n* = 9) or electronic (*n* = 12).	Focus groups, one‐on‐one interviews. Data collection was not reported for every phase. The four focus groups were conducted by the same experienced moderator (female) and trained assistant (female). Data from the interviews were analysed using thematic analysis. A saturation grid was developed to document the concepts endorsed by each participant or focus group
First author: Hayes^15^ Year: 2014 Country: USA	To establish the content validity of two new patient‐reported outcome measures: Sexual Arousal, Interest, and Drive Scale and Hypogonadism Energy Diary.	Hypogonadism (either a prescription for low testosterone treatment or a laboratory sheet showing a total testosterone level < 300 ng/dl(10.4 nmol/L)) No information reported on time since diagnosis	Seventy‐two male participants with a diagnosis of hypogonadism. Note that 90% were older than age 40 years, 63% white, and 93% had acquired hypogonadism as an adult; 40% had high blood pressure, 38% high cholesterol and 15% diabetes; 58% were receiving treatment (unclear if TRT)	Participants were recruited by a recruiting agency primarily through physician referrals and newspaper or internet advertisements between October 2010 and February 2012. Four qualitative studies were done. Only study one was relevant to the current review, which included concept elicitation (i.e., open‐ended questioning to elicit concepts related to experiencing hypogonadism and its treatment). The interviews were scheduled to last 1 h, and the focus groups were 2 h.	Focus groups and individual in‐depth interviews. The same interviewer (male) conducted all focus groups and the interviews. Grounded theory was used. Broad topic areas identification was made. Two independent researchers conducted the analysis.
First author: Rosen^16^ Year: 2009 Country: USA	To develop an instrument that could be used to identify the classification of men with hypogonadism.	Hypogonadal patients (with clinical symptoms of hypogonadism as judged by a physician) and low total testosterone levels. 26 controls, 26 untreated hypogonadism, 26 hypogonadism with TRT. Of those with untreated hypogonadism: Note that 22 of 26 had total testosterone level < 300 mg/dl (10.4 nmol/L); 3 of 26 had testosterone level 300–400 mg/dl (10.4–13.9 nmol/L); 1 of 26 had testosterone level >400 mg/dl (13.9 nmol/L) Months since diagnosis, treated patients = 50.4 (43.1), and untreated = 18.7 (23.3)	Eighty male participants treated (receiving TRT; *n* = 26; mean testosterone 427 [SD 286] ng/dl) and untreated (no TRT in the past 3 months; *n* = 26; testosterone mean 258 [SD 75] ng/dl) diagnosed hypogonadal and eugonadal (control group, *n* = 28) patients from 21 to 74 years old, able to speak and read English, with cognitive competences, and absence of any speech or comprehension difficulties. Patients with any major medical or psychiatric disorder were excluded. 83.7% were white, 10% were Afro‐American, 3.7% were Asian, and 2.5% were Native Hawaiian or other.	Participants were recruited from different sources, including physician providers, community‐based services, health forums and media advertisements. Diagnosed hypogonadal patients (treated and untreated) were recruited from the practices of three physicians who are knowledgeable in the diagnosis and management of hypogonadism. They generated an item pool from focus groups (90‐120 min) and in‐depth interviews (45–90 min). Standardised scoring of the qualitative interviews was used to confirm conceptual domains to generate a questionnaire.	Data collection was through three focus groups (for each of the study groups), including 4–6 patients. Once the recruitment quota for each focus group was met, patients were invited for in‐depth semi‐structured individual interviews. Inductive and deductive approaches and saturation approaches were used. Focus groups and interviews were led by a trained moderator (sex nor reported). Grounded theory was used. Broad topic areas identification. Analysis conducted by two researchers.
First author: Szeinbach^17^ Year: 2012 Country: USA	To create a final conceptual model and the Preference for the testosterone Replacement Therapy (P‐TRT) instrument	Participants agreed to participate in research studies about TRT for conditions associated with a deficiency or absence of endogenous TT. All participants were recruited from a TRT manufacturer's mailing list since they were, or had been, taking TRT for conditions associated with a deficiency or absence of endogenous testosterone. The diagnosis of hypogonadism was not confirmed. In exchange for their participation, participants had the option to accept coupons toward their next purchase of a testosterone replacement therapy product. Gives data on time on TRT – 299 days	Fifty‐eight male participants, mean age 55 [SD 10] years, with current or previous experience using TRT, and be able to receive TRT at the time of the study. Participants used TRT for an average of 175.0 ± 299.2 days. In addition, four participants highlighted having problems with insurance coverage for ART.	Participants were selected from a mailing list containing people who agreed to participate in research studies about TRT for conditions associated with hypogonadism. Enrolment via the online manufacturer‐sponsored website was voluntary. Recruitment took place in December 2011. The instrument development included a literature review, input from expert clinicians and qualitative data. First, a discussion guide was developed from the literature and expert opinion. Next, data was piloted, collected, and coded one‐on‐one from five participant interviews (lasting up to 1 h). Then, one‐on‐one participant interviews (lasting up to 30 min) were conducted using the standard set of questions from the discussion guide. Afterwards, a group of experts (one physician, three researchers with extensive experience in psychometrics, and a nurse practitioner with clinical experience with TRT) tested data and once consensus was reached that all possible items and themes, the final stage included the development of an instrument and conducted in‐depth interviews.	One‐on‐one participant interviews end expert's analysis to create an instrument to conduct in‐depth interviews as part of the cognitive debriefing process. Researchers elicited and recorded responses from participants during interview sessions. Grounded theory was used. Broad topic areas identification. The transcription process included the identification of recurring definitions and themes throughout the text, which produced detailed descriptions and theoretical explanations of the concepts under investigation.
First author: Mascarenhas^18^ Year: 2016 Country: Canada	To explore and describe factors that may influence the rise of prescribing and use of TRT on late‐onset hypogonadism.	Patients TRT users (67% had late‐onset hypogonadism, the rest had different pathologies). Providers included primary care healthcare providers and specialists. Nine patients were recruited. All were on TRT. The diagnosis of hypogonadism was not confirmed. *n* = 6, late‐onset hypogonadism; *n* = 1, HIV; *n* = 1 Klinefelter syndrome; *n* = 1 lymphoma. Years on TRT: Less than 5 = 67%; 5–15 = 22%; and more than 15 = 11%.	Thirteen providers were primary care health providers (three primary care physicians, two nurses, and one pharmacist), and seven were specialists (five urologists and two endocrinologists). All the professionals worked in an urban location, 91% were full‐time health workers, and 47% had >15 years in practice. Nine male participants of >18 years old. Note that 45% of the participants had >65 years old; 55% were full‐time employees, and the rest were unemployed.	All participants (patients and providers) were recruited from Ontario through message distribution (fax, email, social media), clinician networks and circles of contact, posting flyers in clinics. Each interview lasted from 30 to 60 min. The framework approach used and concepts identified from the literature were used to create a guide for the interviews.	Data identified from published? Literature and expert input. One‐on‐one semi‐structured telephone interviews. The Framework approach from Lewis 2003 was used. They developed a coding framework to include topics from raw data and previous concepts. Two analysts independently coded data.

Abbreviations: TRT, testosterone replacement therapy; TT, total testosterone.

### 3.2 Findings

Five broad analytical themes (with several linked descriptive subthemes) were identified from the included studies (Table 1; Table S2) and were ordered according to the decision points that a man with hypogonadism may experience: (1) Symptoms of low testosterone and their impact on daily life; (2) low levels of serum testosterone (consistent with MH); (3) access to treatment information; (4) perceived effects of TRT; (5) expectations, experience, and preference of the type of TRT. Thirteen descriptive interconnected subthemes were identified within these five analytical themes (Table 2).

**TABLE 2:** Thematic analysis of included studies reporting the experience of men with hypogonadism and their healthcare professionals

Theme	Key concepts identified	Sub‐theme (if applicable)	Example quotes
**Low testosterone symptoms and the impact such symptoms have in daily life**	In most of the studies, lack of energy, altered sleeping patterns, lack of strength, weight gain altered sexual activity/desire were the physical symptoms most reported from participants. Emotional/affectional, cognitive and general well‐being effects were also reported. However, the frequency and severity of such symptoms were poorly reported.	**Sexual desire/activity**	I used to feel that I had an extremely active libido, and that went to a very low libido. So, I pretty much didn't initiate any kind of sexual activity. And then even my wife initiated it…" .^16^ "I see stuff, like, I watch a porn video and I don't even get excited. I don't get erect or anything, and that's not like me. . .nothing turns me on" (age 48, adult‐onset).^15^
		**Altered sleeping patterns**	''… mostly, I was just tired. I just didn't have any energy. I just could not—you know what I used to do … I woke up in the morning, I felt like I was more tired than when I went to bed … you just find yourself exhausted. And then on top of it now, I don't have that energy I used to have''.^14^ "Completely exhausted. Could stay in the bed around the clock. Would even put off urinating as long as I could rather than get up and off the bed to go urinate, completely exhausted".^16^
		**Lack of strength**	"Typically, I don't have a hard time falling asleep. I have a hard time staying asleep, in the first hour or so. Typically, if I wake up within the first hour of falling asleep, I'm up for several hours. I can't get myself back to sleep".^16^ "The sleep disturbances the participants described were varied; the participants reported that they regularly woke up at night (*n* = 10; 28 %), had difficulty going back to sleep (*n* = 4; 11%), or had poor quality sleep (*n* = 8; 22 %); nine of the men (25%) reported increased napping".^14^
		**Bodyweight**	"I kept insisting that my weight and my tenderness and everything else wasn't due to over‐eating or over‐drinking or lack of exercise. It was just the opposite. I was working out four days a week. I was running 5 miles. I was playing squash seven days a week. And I was in good shape, but I was getting heavier and heavier… So, I said something is not right." ^16^
		**Perceptions of masculinity**	"Being a man is just being a man. Just, you know. Being alive… Being a man in the sense of… having a good time, keeping your partner happy. Just enjoying life. And that's one part that being a man that I'm not enjoying." ^16^
		**Cognitive function**	"I used to… read a book in two days and tell you everything about it. Can't do that anymore. I do not really want to read a book anymore, because I have to keep going back over and over" .^16^
		**Broader impacts on everyday life and general well‐being**	"Many of the men reported having less confidence or lower self‐esteem (*n* = 10; 28%)." ^14^ "Few men also reported symptoms such as feeling mellow, introversion, feeling alone, fear of rejection, anxiety, and being moody, emotional, or sensitive." ^14^
**The diagnosis of low testosterone and access to treatment information**	Two studies reported patients' perspective regarding getting a diagnosis of HG and the role and relevance of health professionals in this process. However, this information was reported by the authors from the paper rather than from quotes of participants. Szeinbach et al. and Mascarenhas et al. reported that some participants understood the importance of testosterone monitoring and stated it would be easy to get this information from their physicians.		Both patients and providers participants mentioned that they know of primary care physicians or specialists who prescribe TRT without testing for low testosterone levels and based on informal discussions or e‐mail communication" ^18^ "While only two participants were able to recall their testosterone levels, the other three participants understood the importance of testosterone monitoring and stated it would be easy to obtain this information from their physicians." (Authors interpretations ‐Szeinbach et al.)^17^
**Access to treatment information**	Some patients believe that their access to TRT information could facilitate their eventual use. For example, the study in the USA by Szeinbach et al., found that half of the participants described discovering TRT in different ways: either during a consultation with their general practitioner during a session of a related condition or through posters in their pharmacy and health professional practice, though friends and‐workers.		"A couple [of] months ago, [I was] having some blood work done and read an article in Esquire magazine about TT. I asked my family doctor to have that checked". ^18^
**Perceived effects of ART**	Most of the studies reported participants' perceptions of the effects of TRT on different symptoms, which mostly was positive perception towards the improvement of outcomes. However, some participants also reported no effect at all. Across studies, dosages, frequency, and duration of TRT among participants were poorly or not described.	**Sexual desire/activity**	''I have more desire than I did for a long time'' (Participant 01‐108).^14^ ''My energy level's up; my libido's up'' (Participant 01‐109).^14^ ''… the erections were better, sex was better, ejaculations were better; I started noticing a good difference, high energy; I was keeping the weight down'' (Participant 02‐104).^14^
		**Lack of strength**	"Very good. It gives you the energy you need." (ID 16, 62 years old, average TRT use 1460 days ^17^ "…The shots [of TRT] really hype you up, puts you almost on a cocaine buzz." (ID 8, 47 years old, average TRT use 120 days)^17^ "The majority of the participants noticed changes in their energy level and an increased libido after starting testosterone replacement therapy." (Authors interpretation, Gelhorn et al.).^14^
		**Bodyweight**	'''… the erections were better, sex was better, ejaculations were better; I started noticing a good difference, high energy; I was keeping the weight down.''' (Participant 02‐104)^14^
		**Broader impacts on everyday life and general well‐being**	"… one of the biggest benefits [TRT] I get is self‐esteem, because there's more energy and feeling more muscular and masculine. And that goes away when I'm not on the testosterone…" ^16^ "Helped as far as my energy level. I do not know if it has helped with regard to erectile dysfunction, I don't know which part was mental and physical." (ID 7, 54 years old, average TRT use 365 days)^17^
**Expectations, experience, and preference of type of TRT**	One study (Szeinbach et al) was explicitly designed to create a conceptual model and tool to test the Preference for the via??? of administration of TRT among participants. Overall, participants preferred a product that was accessible to use, effortless and comfortable to apply, easy to handle, with accessible application location, and dried quickly.	**Ease of administration**	"The first theme, ease of use, encompassed all topical characteristics associated with testosterone gel products. Participants preferred a product that was convenient to use, easy to apply, easy to handle, with accessible application location, and dried quickly" (Authors interpretations ‐ Szeinbach et al.)^17^
		**Mode of administration**	"I used another product where I had to do the injection into the muscle, and the gel is easier because there is no sticking and blood, and so forth. But the injection more potent; lasts longer." (ID 4, 54 years old, average TRT use 365 days)^17^ "I don't use the gel anymore. I didn't like having to wash my hands every time." [referring to patch TRT]" (ID 9, 55 years old, average TRT use 365 days)^17^
		**Beliefs about effectiveness**:	"… pleased with product; apply by myself; no transportation to doctor's office." [referring to Topical gel TRT]." (ID 1, 48 years old, average TRT use 90 days)^17^ "… Mixed – the gel works and sometimes it does not. My testosterone level has fluctuated, I had had better results with injecting myself, but it is a painful and longer process. Patch leaves giant red marks; topical gel was less robust than injection." (ID 17, 48 years old, average TRT use 1825 days)^17^
		**Perceived adverse effects**:	"I did not like it at all. I was rather annoyed with working with it. Well, I did not like the time that it takes to dry. And then I was running into rash and problems with itching. Never saw results with topical gel." [referring to Topical gel TRT]" (ID 12, 66 years old, average TRT use 90 days)^17^
		**Costs**	"First I found it very expensive; my insurance didn't cover it at all. I did find that it worked fine. I almost liked it better than the shot; it gave me a normal feel. The shots really hype you up, puts you almost on a cocaine buzz" [referring to injection TRT] (ID 8, 47 years old, average TRT use 120 days).^17^

*Note*: Participant details are provided where available.

Abbreviation: TRT, testosterone replacement therapy.

### 3.3 Theme 1: Symptoms of low testosterone and their impact on daily life

As expected, altered sexual desire and/or activity was one of the most frequently cited sub‐themes of low testosterone symptomatology. Some men felt unable to perform sexually in their relationship.^16^ Lack of energ**y** impacted men throughout the day, with some reporting waking up exhausted even after a full night's sleep, and others stating it was worst in the evening. In general, the lack of energy was reported to affect the ability to conduct ‘normal’ daily activities, and men used terms such as ‘tired’, ‘totally exhausted’, ‘lethargic’, ‘sluggish’, and ‘physically drained’ to describe their lack of energy.^15^ Two of the included studies reported that men suffered from sleep disturbances, including falling asleep during the day, night waking, and difficulties going back to sleep.^14^, ^16^ Some men expressed concerns about weight gain and explained that one of the effects of low testosterone was a lack of physical strength, especially concerning those activities they could carry out beforehand.^14^, ^16^ One study interrogated perceptions of masculinity, with men explaining they felt a sense of 'loss of manliness' or 'less of a man', which was implicitly associated with the changes in sexual activity/function.^16^ Low testosterone was described by men to adversely affect their cognitive function, especially memory, concentration, and attention span.^16^ In general, within the cognitive domain, men reported issues with motivation (*n* = 16; 44%), loss of interest (*n* = 11; 31%), memory/forgetfulness (*n* = 11; 31%), focus/concentration (*n* = 6; 17%), drive/ambition (*n* = 3; 8%), attention span (*n* = 3; 8%), and indecisiveness (*n* = 1; 3%).^14^ Men also reported broader impacts on everyday life, general well‐being, and lower mood.

### 3.4 Theme 2: Diagnosis of low testosterone

The authors of two of the included studies reported the participants' experience of getting a diagnosis of hypogonadism.^17^, ^18^ Szeinbach et al. reported that some participants, when asked to recall their testosterone level, recognised the importance of serum testosterone measurement and stated it would be easy to obtain this information from their physicians.^17^ Mascarenhas et al. discussed the persistence of some participants, defined as 'drug seekers', to acquire and use TRT, irrespective of the advice of their physicians.^18^ These 'drug seekers' were reported to have consulted multiple physicians to get a prescription (regardless of the diagnosis). Mascarenhas et al. also reported that one participant took the liberty of increasing his TRT dose and, when he failed to perceive any immediate effects, requested switching TRT products.^18^

### 3.5 Theme 3: Access to treatment information

Participants reported that access to information about TRT was an important factor determining their eventual use of TRT. For example, Szeinbach et al. observed that participants received TRT via different routes: During a consultation (e.g., with their general practitioner regarding a related condition), through posters at their pharmacy, through friends and co‐workers, popular magazines, and Internet searching.^17^ Mascarenhas et al. reported that some participants expressed the desire to receive more information on the advantages and risks of TRT from their physicians and explained that ‘while most participants find it easy to access information on the positive effects of TRT and how to acquire it, they seem to have little knowledge about its side effects or risks’; they also pointed out that participants felt that the marketing and advertisements 'spoke to' their perceived needs.^18^ Information on improved sexual function and energy levels was of greatest interest to participants, whereas information concerning the side effects of TRT was sought to a much lesser extent.

### 3.6 Theme 4: Perceived effects of TRT

In three studies,^14^, ^15^, ^16^, ^17^ participants described how TRT positively impacted their sexual desire/activity, while in one study, some participants did not experience any significant improvements. Participants from three of the included studies discussed their experience of improvements in strength/energy while receiving TRT. In one study, participants described an 'energy boost' after TRT.^17^ Some participants observed positive changes in body shape and increased muscle bulk. Participants commented positively on the improved energy levels throughout the day. However, some participants did not achieve the expected impact of TRT on energy levels. One man experienced weight loss as a positive outcome of TRT.^14^ Three studies reported positive impacts on general well‐being.^14^, ^16^, ^17^ Szeinbach et al. reported that some participants experienced general well‐being changes, often described as ‘I feel like myself again’.^17^ One man described a positive change in self‐esteem as a result of being more energetic and masculine.^16^ Another man recognised that not all outcomes improved after TRT and acknowledged that some experienced benefits could be interrelated. Another man reported a broader range of symptoms and recognised the relatedness and interplay between them.^16^ Some of these symptoms included psychological (e.g., anxiety), emotional (e.g., self‐esteem), or well‐being (e.g., masculinity perceptions) outcomes that were reported as improved after the therapy.

### 3.7 Theme 5: Expectations, experience, and preference of the type of TRT

Three studies reported participants' expectations, experience, and preference about TRT type. Five sub‐themes were identified across the included studies, relating to ease of administration, mode of administration; beliefs about effectiveness; perceived adverse effects; and costs. One study was designed to create a conceptual model and tool to test participants' preference for ease of administration of TRT.^17^ This study assessed the experiences and perceptions of participants for different types of TRTs (i.e., gel vs. injections vs. patches). Overall, participants expressed their preference for a product that was ‘accessible to use’, ‘effortless’, ‘comfortable to apply’, and ‘easy to handle’. In two studies, participants reported varied perspectives about the mode of administration of the TRT; preferences were highlighted for crucial features of the route of delivery, which were linked back to ease of administration and perceptions about effectiveness. Only one of the included studies reported the participants' beliefs about effectiveness concerning different types of TRT. Two studies reported participants' concerns about perceived adverse effects associated with the TRT. One study described specific problems such as rashes, itching, or pain after administration (referring to TRT injections). One study reported that the cost of treatment was among the factors considered by participants when expressing their preferences for TRT products. Some participants described how features of their insurance plans (e.g., co‐pay help programmes to top up the cost of the preferred treatment) influenced their choice of treatment.

### 3.8 Quality assessment

The methodological quality of the five included studies was assessed using the CASP tool (Table S3). As the included studies sought to interpret or illuminate the actions and/or subjective experiences of the recruited participants, their findings were considered valid and relevant to address the research question of this qualitative synthesis. The research design varied across studies. Apart from Mascarenhas et al., all studies justified and rationale for choosing their study design.^18^ Documenting recruitment strategy and clinical setting are important to identify potential selection bias, these were explained in all studies except for Gelhorn et al.^14^ Three studies provided information on the relationship between the researchers and the participants^15^, ^16^, ^17^; for the remaining two studies, the researchers did not critically assess their role and potential influence during the study.^14^, ^18^ The study by Gelhorn et al. was considered at potential risk of bias as it was sponsored by a pharmaceutical company that remunerated some of the authors.^14^ The funder's role in the analysis of data or presentation of conclusions was not reported. All the studies discussed the contribution of their findings to existing knowledge or understanding.

### 3.9 Confidence in the findings

GRADE‐CERQual assessment was used to assess confidence in the themes/subthemes identified in this qualitative synthesis (Table 3). Moderate confidence was expressed for 16 themes/subthemes and low confidence for four. None of the qualitative evidence received a high confidence judgement. Findings were downgraded for lack of reported researcher reflexivity (e.g., failing to acknowledge potential sponsor bias), adequacy of data, or poor reporting of participants' sociodemographic characteristics.

**TABLE 3:** Confidence in Evidence from Reviews of Qualitative research (CERQual) evidence profile

**Summary of review finding**	**Studies contributing to review finding**	**Methodological limitations**	**Coherence**	**Adequacy**	**Relevance**	**CERQual assessment of confidence in the evidence**
**Theme 1: Symptoms of low testosterone and impacts on daily life**						
1. Altered sexual desire/activity	Gelhorn et al.^14^,^27^; Hayes et al.^15^; Rosen et al.^16^	**Moderate** concerns about methodological limitations, one study did not adequately address the recruitment strategy or analysis.	**No concerns** about coherence	**Minor concerns** about adequacy. Three studies offered moderately rich data. Data retrieved come from direct participants quotes and some from authors' interpretation.	**Moderate concerns** about relevance given that most included population were white.	**Moderate confidence**
2. Lack of energy	Gelhorn et al.^14^,^27^; Hayes et al.^1^; Rosen et al.^16^	**Moderate** concerns about methodological limitations, one study did not adequately address the recruitment strategy or analysis.	**No concerns** about coherence	**Minor concerns** about adequacy. Three studies offered moderately rich data. Data retrieved come from direct participants quotes and some from authors' interpretation.	**Moderate concerns** about relevance given that most included population were White.	**Moderate confidence**
3. Lack of strength	Gelhorn et al.^14^,^27^; Rosen et al.^16^	**Moderate** concerns about methodological limitations.	**Minor concerns** about coherence. Some data slightly ambiguous.	**Moderate concerns** about adequacy. Two studies offered relatively limited data. Data retrieved come from direct participants quotes and some from authors' interpretation.	**Moderate concerns** about relevance given that most included population were White.	**Moderate confidence**
4. Altered sleeping patterns	Gelhorn et al.^14^,^27^; Rosen et al.^16^	**Moderate** concerns about methodological limitations.	**Minor concerns** about coherence. Some data slightly ambiguous.	**Moderate concerns** about adequacy. Two studies offered relatively limited data. Data retrieved come from direct participants quotes and some from authors' interpretation.	**Moderate concerns** about relevance given that most included population were White.	**Moderate confidence**
5. Weight gain	Gelhorn et al.^14^,^27^; Rosen et al.^16^	**Moderate** concerns about methodological limitations.	**Minor concerns** about coherence. Some data slightly ambiguous.	**Moderate concerns** about adequacy. Two studies offered relatively limited data. Data retrieved come from direct participants quotes and some from authors' interpretation.	**Moderate concerns** about relevance given that most included population were White.	**Moderate confidence**
6. Perceptions of masculinity	Rosen et al.^16^	**No concerns** about methodological limitations.	**No concerns** about coherence.	**Moderate concerns** about adequacy because of relatively limited data.	**Moderate concerns** about relevance given that most included population were White.	**Moderate confidence**
7. Cognitive function	Gelhorn et al.^14^; Hayes et al.^15^; Rosen et al.^16^	**Moderate** concerns about methodological limitations, one of the studies did not adequately address the recruitment strategy or analysis.	**No concerns** about coherence.	**Moderate concerns** about adequacy. Two studies offered relatively limited data. Data retrieved come from direct participants quotes and some from authors' interpretation.	**Moderate concerns** about relevance given that most included population were White.	**Moderate confidence**
8. Broader effects on everyday life	Gelhorn et al.^14^,^27^; Hayes et al.^15^; Rosen et al.^16^	**Moderate** concerns about methodological limitations, one study did not adequately address the recruitment strategy or analysis.	**No concerns** about coherence.	**Moderate concerns** about adequacy. Two studies offered relatively limited data. Data retrieved come from direct participants quotes and some from authors interpretation.	**Moderate concerns** about relevance given that most included population were White.	**Moderate confidence**
**Theme 2: Diagnosis of hypogonadism**						
9. Diagnosis of low TT	Szeinbach et al.^17^; Mascarenhas et al.^18^	**Moderate concerns** about methodological limitations, one study was overall poor quality.	**Minor concerns** about coherence. Some data slightly ambiguous.	**Moderate concerns** about adequacy. Two studies offered relatively limited data. Data retrieved come from authors interpretation.	**Significant concerns** about relevance. Neither study reported ethnicity.	**Low confidence**
**Theme 3: Access to treatment information**						
10. Access to treatment information	Mascarenhas et al.^18^	**Significant concerns** about methodological limitations, included study was overall poor quality.	**No concerns** about coherence.	**Moderate concerns** about adequacy. Offered relatively limited data with most data from author's interpretation.	**Significant concerns** about relevance. Study did not report ethnicity.	**Low confidence**
**Theme 4: Perceived effects of testosterone replacement therapy**						
11. Sexual desire/activity outcomes	Gelhorn et al.^14^,^27^; Hayes et al.^15^; Rosen et al.^16^	**Moderate** concerns about methodological limitations, one of the studies did not adequately address the recruitment strategy or analysis. (Reflexivity was not addressed in the two studies, which may be particularly important given funded by the pharmaceutical industry)	**No concerns** about coherence.	**Minor concerns** about adequacy. Three studies offered moderately rich data. Data retrieved come from direct participants quotes and some from author's interpretation.	**Moderate concerns** about relevance given that most included population were White.	**Moderate confidence**
12. Strength/Energy outcomes	Gelhorn et al.^14^,^14^; Rosen et al.^16^; Szeinbach et al.^17^	**Moderate** concerns about methodological limitations, one study did not adequately address the recruitment strategy or analysis. (Reflexivity was not addressed in one study, which may be particularly important given funded by the pharmaceutical industry)	**No concerns** about coherence.	**Minor concerns** about adequacy. Three studies offered moderately rich data. Data retrieved come from direct participants quotes and some from authors interpretation.	**Moderate concerns** about relevance given that most included population were White, and one study did not report ethnicity.	**Moderate confidence**
13. Weight loss	Gelhorn et al.^14^,^27^	**Moderate** concerns about methodological limitations did not adequately address the recruitment strategy or analysis.	**No concerns** about coherence.	**Moderate concerns** about adequacy because of limited data.	**Moderate concerns** about relevance given that most of the included population were White.	**Moderate confidence**
14. Emotional/affectional/well‐being outcomes	Rosen et al.^16^	**No concerns** about methodological limitations.	**No concerns** about coherence.	**Moderate concerns** about adequacy because of limited data.	**Moderate concerns** about relevance given that most included population were White.	**Moderate confidence**
15. Cognitive function outcomes	Gelhorn et al.^14^,^27^; Rosen et al.^16^	**Moderate concerns** about methodological limitations.	**Minor concerns** about coherence. Some data slightly ambiguous.	**Moderate concerns** about adequacy because of limited data.	**Moderate concerns** about relevance given that most included population were White.	**Moderate confidence**
16. General well‐being outcomes	Szeinbach et al.^17^	**Minor concerns** about methodological limitations.	**No concerns** about coherence.	**Moderate concerns** about adequacy because of limited data.	**Moderate concerns** about relevance. Study did not reported ethnicity.	**Moderate confidence**
17. Ease of administration	Szeinbach et al.^17^	**Minor concerns** about methodological limitations.	**Minor concerns** about coherence. Some data slightly contradictory.	**Moderate concerns** about adequacy because of limited data.	**Moderate concerns** about relevance. Study did not reported ethnicity.	**Moderate confidence**
18. Perceived adverse effects	Szeinbach et al.^17^; Mascarenhas et al.^18^	**Moderate concerns** about methodological limitations, one study was overall poor quality.	**Minor concerns** about coherence. Some data slightly contradictory.	**Moderate concerns** about adequacy. Two studies offered relatively limited data. Data retrieved come from authors interpretation.	**Significant concerns** about relevance. Neither study reported ethnicity.	**Low confidence**
19. Beliefs about effectiveness	Szeinbach et al.^17^	**Minor concerns** about methodological limitations.	**Minor concerns** about coherence. Some data slightly contradictory.	**Moderate concerns** about adequacy because of limited data.	**Moderate concerns** about relevance. Study did not report ethnicity.	**Moderate confidence**
20. Mode of administration	Hayes et al.^15^; Szeinbach et al.^17^	**Moderate concerns** about methodological limitations.	**Minor concerns** about coherence. Some data contradictory.	**Minor concerns** about adequacy. One study offered relatively limited data. Data retrieved come from direct participants quotes and some from author's interpretation.	**Moderate concerns** about relevance. Only one study reported ethnicity, and most of the participants were White.	**Moderate confidence**
21. Costs	Szeinbach et al.^17^	**Minor concerns** about methodological limitations.	**No concerns** about coherence.	**Moderate concerns** about adequacy because of limited data.	**Moderate concerns** about relevance. Study did not report ethnicity.	**Moderate confidence**

## 4 DISCUSSION

There exists high‐quality evidence that MH is associated with an increased risk of sexual symptoms.^5^ However, men often experience a multitude of functional symptoms and other impairments of well‐being, which clinicians often dismiss because the evidence is less clear for their effective treatment by TRT.^2^ Increased regulatory importance is being placed on establishing the efficacy of drugs for MH by measuring outcomes important to patients because they provide direct evidence of how patients feel or function.^11^ Herein, we summarise the evidence for how men experience hypogonadism, TRT, and the impacts on their lives. Our analysis is based on limited data but suggests that hypogonadism may impact several physical and mental well‐being aspects, many of which are not captured sufficiently by prior RCTs. We also highlight that the extents to which cultural, ethnic, geographic and socioeconomic factors influence the experience of MH are largely unknown.

**FIGURE 2:**
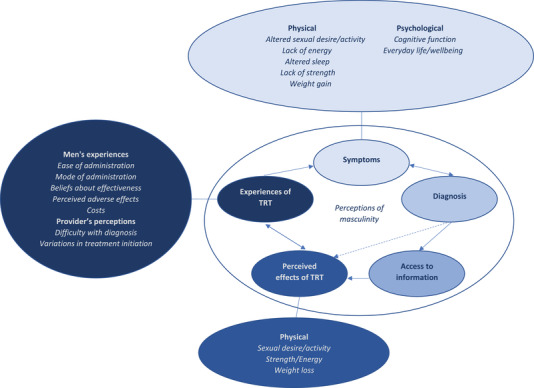
Conceptual diagram of the evidence synthesis. Abbreviation: TRT, testosterone replacement therapy

Functional symptoms such as tiredness and reduced cognition may arise for many reasons other than MH, particularly when combined with co‐morbidities.^19^ Our analysis, therefore, excluded studies that restricted the reporting of specific (sexual) symptoms. We also excluded studies restricted to subtypes of MH (e.g., androgen deprivation therapy for prostate cancer) due to the risks of conflating the experience of MH with other conditions. While reducing the pool of data included, our analysis is strengthened by synthesising available evidence for how MH per se impacts men. Our evidence synthesis included studies of varying methods and scope; data were identified and organised according to the different key stages and decision points that a man with hypogonadism encounters from diagnosis to the point of treatment. However, this process might not be linear, with some men circling back to seek additional information if the perceived effectiveness of one type of TRT has not been met, and some men might not experience all the phases, with certain physicians even proceeding straight to TRT without having performed any specific diagnostics (Figure 2).

Sexual dysfunction is by far the most consistently reported symptomatic outcome reported in quantitative studies of MH.^2^ Consistent with this, sexual desire/activity was a commonly reported sub‐theme by participants across two analytical themes (i.e., symptoms of low testosterone and impacts on daily life; the perceived effects of TRT). Our analysis is in concordance with a review of the experience of sexual symptoms in men with MH.^20^ Our analysis suggested that some men with MH may experience sleep disturbances, lack of physical strength, reduced cognitive function, lower mood and broader impacts on everyday life, and general well‐being for which individual studies have yielded contradictory or equivocal results.^2^, ^21^ Our analysis also identified that some participants with MH may experience an adverse impact on perceptions of masculinity, which has been reported previously.^22^, ^23^ Altered perceptions of masculinity have also been reported to change the way men may experience and seek help for other health issues such as depression.^22^, ^23^

As reported previously, some examples of men's and physicians' behaviour described in these studies may lead to unnecessary prescribing of TRT.^24^, ^25^ For instance, the described ‘testosterone‐seeking’ attitude (wherein men sought new medical opinions until one eventually agreed to prescribe), along with the tendency of certain physicians to ascribe a broad generality of symptoms to ‘low testosterone’ and thus prescribe TRT with no prior meaningful diagnostics. This qualitative synthesis suggests that physician knowledge, experience, and preferences may impact the extent to which men might ascribe their symptoms to low testosterone level (or make alternative associations) and, hence, affect their expectations of what TRT might realistically achieve for them. Furthermore, data from the current study suggest that men with hypogonadism may require a more coherent, holistic narrative of their condition from their physicians that is not broken down into disconnected chunks labelled ‘sexual function’, ‘mental health’, or ‘physical performance’.

Our analysis is limited by only having available published data from North American studies. There are likely to be important differences between US‐based, privately funded clinics specialising in 'low testosterone', and European‐based endocrinology or andrology units in public hospitals. Therefore, the current analysis findings may not be broadly applicable outside North America. Most of the studies provided quotes directly from the participants to support the identification of specific themes/subthemes; however, some studies provided only the authors' interpretations. The quality of the five included studies according to the CASP tool showed that the results across studies were valid and relevant to the scope of this qualitative synthesis. However, the small number of identified studies that provided in‐depth data directly from the participants is a limitation of this work. In two of the included studies,^17^, ^18^ the diagnostic criteria for MH were not specified, so it was assumed that only participants were given TRT following appropriate clinical and biochemical assessments. Furthermore, information on the frequency of symptoms and characteristics of TRT (i.e., type, dose, route of administration, frequency of use) were poorly reported across included studies.

We excluded any study restricted to a single aetiology of hypogonadism, that is, reporting on specific named conditions or diseases associated with hypogonadism, such as men with Klinefelter's syndrome, congenital hypogonadal syndromes, or receiving androgen‐deprivation therapy for prostate cancer. The rationale for this was to avoid the confounding effect of symptoms arising directly from aspects of these conditions that are unconnected to hypogonadism. While this approach taken in our analysis led to a smaller number of included studies, loosening the inclusion criteria to encompass these may have paradoxically weakened our conclusions.

In summary, we acknowledge that the current study is based on limited evidence; nevertheless, it provides a framework of evidence that mirrors core aspects of the pragmatic experience of patients. Many facets of the MH experience are unaddressed and thought untreatable by clinicians. Symptoms such as tiredness, reduced cognition, and/or reduced muscle strength may not be thought consequential to MH in some patients; however, it is beyond doubt men with hypogonadism commonly experience them and therefore warrants treatment (endocrine or otherwise). Based on the current study, we make three recommendations. Firstly, some men with MH may benefit from a holistic, patient‐centred approach to improving well‐being and quality of life, rather than the traditional focus on discreet symptoms (often sexual) practised by most clinicians. Secondly, the experience of men with MH is likely to be profoundly influenced by cultural identity and background, but our study reveals that this hypothesis remains unexplored; studying the impact of MH on men within different populations could improve the targeting of information and treatment monitoring for under‐served demographic groups.^26^ Finally, further research is needed to determine what resources clinicians require to support men with less specific hypogonadal symptoms with regard to accessing unbiased, patient‐focused educational resources. Such future approaches would have the potential to impact healthcare quality for men with hypogonadism positively.

## CONFLICT OF INTEREST

C.N.J. received honorarium for debate on testosterone use organised by Society for Endocrinology, sponsored by Besins Healthcare. R.Q. received a speaker honorarium and conference support from Bayer UK. M.A.‐M., M.B., M.C., P.M., J.H., N.O., R.H., L.A., F.W., W.S.D., S.B., and K.G. have no conflict of interest.

## AUTHOR CONTRIBUTIONS

Magaly Aceves‐Martins, Miriam Brazzelli, Moira Cruickshank, Channa N Jayasena, and Katie Gilles had substantial contributions to the research design, data analysis, or interpretation. P.M. was key for the searches and data acquisition. Richard Quinton, Jemma Hudson, Nick Oliver, Rodolfo Hernandez, Lorna Aucott, Frederick Wu, Waljit S. Dhillo, and Siladitya Bhattacharya had a substantial contribution in the interpretation of data. All the authors revised the review critically. All the authors approved the submitted version.

## Supporting information



Supporting information
