# Supplementary material for: Identifying the outcomes important to men with hypogonadism: A qualitative evidence synthesis
Source: Andrology. 2022 Feb 8;10(4):625–41. doi: 10.1111/andr.13156 (PMC9487983; doi:10.1111/andr.13156)
Supplement: Supplementary file 1 — Supporting information [file ANDR-10-625-s001.docx]

**Appendix 1.**

**Search strategies**

**Identifying the outcomes important to men with hypogonadism: a qualitative evidence synthesis**

**Search strategies**

**Ovid Embase, Ovid MEDLINE(R) and Epub Ahead of Print, In-Process & Other Non-Indexed Citations and Daily**

1 exp androgens/tu use ppez

2 hormone replacement therapy/ use ppez

3 2 and (men or androgen? or testosterone).af.

4 Androgen Therapy/ use emez

5 androgen replacement therapy.tw,kw.

6 testosterone.tw,kw.

7 or/1,3-6

8 exp Erectile Dysfunction/ use ppez

9 exp impotence/ use emez

10 Sexual Dysfunction, Physiological/

11 testosterone/df

12 Libido/ use ppez

13 Libido Disorder/ use emez

14 Hypogonadism/

15 (erectile adj3 dysfunction).tw,kw.

16 (libido adj3 (low$ or decreas$ or reduc$ or loss)).tw,kw.

17 (impotence or impotent).tw,kw.

18 hypogonad$.tw,kw.

19 (low$ adj3 testosterone).tw.

20 (deficien$ adj3 (androgen or gonad$ or testosterone)).tw.

21 (insuffic$ adj3 (androgen or gonad$ or testosterone)).tw.

22 (kallman or klinefetter).tw.

23 or/8-22

24 qualitative research/

25 qualitative research.tw,kw.

26 (qualitative adj3 method$).tw.

27 (qualitative method? or qualitative methodology).kw.

28 (qualitative adj3 stud$).tw.

29 qualitative study.kw.

30 focus groups/ use ppez

31 focus group?.tw,kw.

32 grounded theory/

33 grounded theory.tw,kw.

34 narrative analys?s.tw,kw.

35 process evaluation.tw,kw.

36 mixed method?.tw,kw.

37 mixed method$.mp.

38 mixed methodology.tw,kw.

39 (in depth adj4 interview$).tw.

40 in depth interview?.kw.

41 ((semi structured or semistructured) adj5 interview$).tw.

42 semi structured interview?.kw.

43 qualitative interview$.tw.

44 qualitative interview?.kw.

45 (interview$ and theme$).tw.

46 interview?.kw.

47 (interview$ and audio recorded).tw.

48 qualitative case stud$.tw.

49 descriptive case stud$.tw.

50 qualitative case study.kw.

51 descriptive case study.kw.

52 qualitative exploration.tw,kw.

53 qualitative evaluation.tw,kw.

54 qualitative intervention.tw,kw.

55 qualitative approach.tw,kw.

56 qualitative inquiry.tw,kw.

57 qualitativ$ analys$.tw.

58 qualitative analysis.kw.

59 (qualitative adj3 data).tw.

60 qualitative data.kw.

61 discourse analysis.tw,kw.

62 discursive.tw,kw.

63 phenomenological.tw,kw.

64 thematic analysis.tw,kw.

65 ethnograph$.tw.

66 ethnography.kw.

67 action research.tw,kw.

68 ethno?methodology.tw,kw.

69 social construction.tw,kw.

70 or/24-69

71 phenomenological characteristics.tw,kw.

72 phenomenological model.tw,kw.

73 action research arm test.tw,kw.

74 protocol.ti.

75 or/71-74

76 70 not 75

77 7 and 76

78 23 and 76

79 77 or 78

80 exp animals/ not human/

81 exp nonhuman/ not humans/

82 79 not (80 or 81)

83 82 and male/

84 82 not ((women not men) or (female not male)).tw.

85 83 or 84

86 limit 85 to yr="1992 -Current"

**Ovid PsycINFO**

1 hormone therapy/

2 1 and (men or androgen? or testosterone).af.

3 androgen replacement therapy.tw.

4 testosterone.tw.

5 2 or 3 or 4

6 erectile dysfunction/

7 libido/ or sex drive/

8 hypogonadism/

9 (erectile adj3 dysfunction).tw.

10 (libido adj3 (low$ or decreas$ or reduc$ or loss)).tw.

11 (impotence or impotent).tw.

12 hypogonad$.tw.

13 (low$ adj3 testosterone).tw.

14 (deficien$ adj3 (androgen or gonad$ or testosterone)).tw.

15 (insuffic$ adj3 (androgen or gonad$ or testosterone)).tw.

16 or/6-15

17 qualitative research/

18 qualitative research.tw.

19 (qualitative adj3 method$).tw.

20 (qualitative adj3 stud$).tw.

21 focus group?.tw.

22 grounded theory/

23 grounded theory.tw.

24 narrative analys?s.tw.

25 process evaluation.tw.

26 mixed method?.tw.

27 mixed methodology.tw.

28 (in depth adj4 interview$).tw.

29 ((semi structured or semistructured) adj5 interview$).tw.

30 qualitative interview$.tw.

31 (interview$ and theme$).tw.

32 interview?.kw.

33 (interview$ and audio recorded).tw.

34 qualitative case stud$.tw.

35 descriptive case stud$.tw.

36 qualitative exploration.tw.

37 qualitative evaluation.tw.

38 qualitative intervention.tw.

39 qualitative approach.tw.

40 qualitative inquiry.tw.

41 qualitativ$ analys$.tw.

42 (qualitative adj3 data).tw.

43 discourse analysis/

44 discursive.tw,kw.

45 phenomenological.tw.

46 thematic analysis.tw.

47 ethnograph$.tw.

48 action research.tw.

49 ethno?methodology.tw.

50 social construction.tw.

51 or/17-50

52 phenomenological characteristics.tw.

53 phenomenological model.tw.

54 action research arm test.tw.

55 protocol.ti.

56 or/52-55

57 51 not 56

58 5 and 57

59 16 and 57

60 58 or 59

61 limit 60 to yr="1992 -Current"

**EBSCO CINAHL**

S19 S8 AND S17

Limiters - Published Date: 19920101-

S18 S8 AND S17

S17 S9 OR S10 OR S11 OR S12 OR S13 OR S14 OR S15 OR S16

S16 TX discourse analysis OR TX discursive OR TX thematic analysis OR TX ethnography OR TX action research OR TX phenomenological

S15 TX qualitative exploration OR TX qualitative evaluation OR TX qualitative intervention* OR TX qualitative approach OR TX qualitative analysis OR TX qualitative data

S14 TX mixed method* OR TX semi structured interview* OR TX in depth interview*

S13 TX focus group* OR TX grounded theory OR TX narrative analysis

S12 TX qualitative n3 research OR TX qualitative n3 method* OR TX qualitative n3 study

S11 (MH "Focus Groups")

S10 (MH "Semi-Structured Interview") OR (MH "Structured Interview") OR (MH "Narratives")

S9 (MH "Qualitative Studies+")

S8 S1 OR S2 OR S3 OR S4 OR S5 OR S6 OR S7

S7 TX ( (deficien* N3 (androgen or gonad* or testosterone)). ) OR TX ( (insuffic* adj3 (androgen or gonad* or testosterone)). )

S6 ( (libido N3 (low* or decreas* or reduc* or loss) ) OR hypogonad* OR low* N3 testosterone

S5 TX erectile N3 dysfunction OR TX impotence OR TX impotent

S4 (MH "Sexual Dysfunction, Male")

S3 (MH "Hypogonadism+")

S2 (MH "Impotence")

S1 (MH "Testosterone Replacement Therapy") OR TX androgen replacement therapy OR TX testosterone

**ProQUEST ASSIA**

(((MAINSUBJECT.EXACT("Testosterone") OR MAINSUBJECT.EXACT("Hormone replacement therapy")) OR (androgen replacement therapy) OR ((hypogonadism or impotence or impotent ) OR (erectile W3 dysfunction)) OR ((libido w3 (low* or decreas* or reduc* or loss)) OR (low* w3 testosterone)) OR ((deficien* W3 (androgen or gonad* or testosterone)) OR (insuffic* W3 (androgen or gonad* or testosterone)))) AND (men OR male)) AND (MAINSUBJECT.EXACT("Qualitative research") OR (qualitative OR focus group* OR interview* OR mixed method* OR ethnography OR phenomenological OR discourse analysis OR discursive)
